# The impact of film audio description style on presence in Chinese visually impaired audiences

**DOI:** 10.3389/fpsyg.2025.1541539

**Published:** 2025-05-21

**Authors:** Hui Yang, Danni Xie, Yutao Li

**Affiliations:** ^1^School of Foreign Languages, Lanzhou Jiaotong University, Lanzhou, China; ^2^Lanzhou Library, Lanzhou, China

**Keywords:** audio description, presence, subjective, objective, Chinese visually impaired

## Abstract

This study investigates the impact of film audio description (AD) styles on dimensions of presence—spatial presence, engagement, ecological validity, and negative effects—among visually impaired audiences in China. Two distinct styles of AD were evaluated: objective AD and subjective AD. Using a mixed-methods methodology that integrated quantitative surveys with qualitative focus group discussions, the study examined participants’ emotional and perceptual responses. The findings indicated that the subjective AD style significantly enhanced key dimensions of presence, including greater engagement, improved spatial awareness, and increased ecological validity while reducing confusion compared to the objective style. These effects were consistent across participants with varying levels of visual impairment and educational backgrounds, demonstrating the broad applicability of the subjective AD style. The study underscores the potential of subjective AD to enhance immersion and elevate the viewing experience, contributing to more inclusive and effective media accessibility for visually impaired audiences.

## 1 Introduction

Audio Description (AD) is a crucial accessibility tool that enables visually impaired audiences to engage with visual media. However, the style of AD can significantly influence how users perceive, interpret, and immerse themselves in audiovisual content. This study investigates two distinct AD styles—objective and subjective—and their impact on the sense of presence, a critical dimension of media immersion.

In this study, objective AD is defined as a neutral and factual approach that aims to describe only what is visually observable, avoiding interpretation or emotional coloring. This style aligns with what Kleege (2016, p. 96) terms “a form of surface reading where the emphasis is on what’s manifest.” It primarily serves a referential function, delivering information clearly and efficiently (Bartolini and Manfredi, 2022). In contrast, subjective AD is defined as an interpretive and emotionally expressive style that goes beyond surface-level description. It incorporates figurative language, evaluative commentary, and inferences about character emotions or narrative tone. This style not only describes visual elements but also conveys the describer’s subjective judgments and personal comments.

While both styles serve important communicative functions, cultural context plays a pivotal role in shaping their appropriateness and effectiveness. In Western countries such as the United Kingdom and the United States, objective AD is often regarded as the professional norm, reflecting a preference for neutrality and factual accuracy. However, in Chinese AD practice, there has been a notable tendency toward subjective AD, often characterized by interpretive elaboration and evaluative language. Such stylistic tendencies highlight the importance of exploring how Chinese audiences perceive and respond to different AD styles.

Although prior research has highlighted the importance of presence in improving media accessibility, systematic comparisons of these AD styles remain limited, particularly in the context of Chinese visually impaired audiences.

This study addresses this gap by focusing on visually impaired individuals in China, a demographic often underrepresented in accessibility research. It explores how subjective AD influences key dimensions of presence, including emotional engagement, spatial awareness, and ecological validity compared to its objective counterpart. Using a mixed-methods design that combines quantitative analysis with qualitative insight and focusing on the experiences of Chinese visually impaired audiences, this study provides novel insights into enhancing media accessibility in China. It also underscores the potential of subjective AD to foster more immersive and inclusive viewing experiences.

## 2 Literature review

### 2.1 Objectivity and subjectivity in audio description

As a form of inter-semiotic translation, AD aims to “provide access for the blind and partially sighted audiences” (Bardini, 2020a, p. 274) by “translating visual images into verbal descriptions” (Braun, 2008, p. 14). When creating ADs for films, three key questions arise: “What should be described? When should the description be given? And how should the visual and aural information be described?” (Vercauteren, 2007, p. 142). To address these questions and standardize the creation of AD, many countries have developed AD guidelines (France: Morisset and Gonant, 2008; Germany: Benecke and Dosch, 2004; Greece: Georgakopoulou, 2008; USA: American Council of the Blind, 2009). Most AD guidelines advocate for an objective approach (ADC, 2009; AENOR, 2005; ITC, 2000; Morisset and Gonant, 2008; Salzhauer Axel et al., 2003; Snyder, 2010).

For many years, objectivity has been regarded as a fundamental quality standard in AD, and it remains a core principle reflected in numerous AD guidelines. The American Council of the Blind (2009) emphasize that “The best audio describers objectively recount the visual aspects of an image. Subjective or qualitative judgments or comments get in the way—they constitute an interpretation on the part of the describer and are unnecessary and unwanted.” *The ITC Guideline On Standards For Audio Description* (ITC, 2000) specifies “Describe what is there, do not give a personal version of what is there.” Similarly, *The Ultimate Guide to Audio Description* (3Play Media, n.d.) recommends “Describe what you see without interpretation or personal comment.”

The primary motivation behind the emphasis on objectivity is to respect users’ autonomy, leaving sufficient room for AD users to form their mental images of the content independently (Schaeffer-Lacroix et al., 2023, p. 2). However, this rationale is increasingly questioned, as visually impaired individuals do not have the same visual and informational access as sighted viewers. They often rely on alternative tools and methods for accessing information, with many preferring more multisensory approaches to engage with visual content (Luque Colmenero, 2024, p. 40). Furthermore, achieving complete objectivity in AD is not always feasible. Udo and Fels (2009, p. 179) argued that objective interpretation was impossible, while Hyks (2005) acknowledged that AD was inherently subjective, as individuals saw and expressed things differently. The Conseil Supérieur de L’Audiovisuel (2020) also asserted that objectivity could not be fully attained. In April 2024, Ofcom, the UK’s media regulator and a pioneer in publishing widely used accessibility guidelines updated their guidelines, and proposed “to remove the recommendation that AD should be ‘unobtrusive and impersonal’ and instead encourage providers to consider different approaches to AD (such as more traditional or creative styles), taking account of their given audience’s needs and preferences, and the genre of the programme” (Ofcom, 2024).

Emerging evidence suggests that subjective descriptions may offer significant benefits for specific segments of the visually impaired population (Kruger, 2010; Orero and Vercauteren, 2013; Palmer and Salway, 2015; Fresno et al., 2016; Geerinck and Vercauteren, 2020). Currently, “a consensus exists that a level of subjectivity is part of the AD process given that interpretation is always required and is essentially individual” (Schaeffer-Lacroix et al., 2023, p.2). The investigation of subjectivity in AD has become a focal point within reception studies. Several studies (Fryer and Freeman, 2012a; Szarkowska, 2013; Ramos Caro, 2016; Walczak and Fryer, 2017; Bardini, 2020b; Luque Colmenero and Soler Gallego, 2020; Soler Gallego and Luque Colmenero, 2022; Soler Gallego and Luque Colemero, 2023) have explored AD styles that deviate from the objective approach. Research in theatrical contexts suggested that audiences generally responded positively to subjective ADs (Udo and Fels, 2009; Udo et al., 2010). In the cinematic domain, empirical findings indicated that creative ADs could significantly enhance the sense of presence, thereby fostering a more immersive viewing experience (Walczak and Fryer, 2017). Ramos Caro (2016) examined the emotional reception of objective versus subjective ADs and concluded that descriptions incorporating emotionally charged language elicited stronger emotional responses from audiences. Fresno (2014) found that semantic AD enhanced information retention compared to traditional AD methods. Luque Colmenero (2024, p. 49) argued that “different styles of AD, including metaphorical, immersive, synesthetic, interpretive voices, and poetic styles, have the potential to enhance the subjective and aesthetic experience of museum visitors.”

The study of AD reception in China remains in its early stages, with limited literature available on the topic. Xiao and Dong (2020) conducted an experiment and survey with visually impaired individuals in Shanghai, comparing artificial-voice AD to human-voice AD, and found a preference for human voices. Shen and Lei (2021) explored AD interpreting techniques through the practical operation of the Hangzhou barrier-free film project. Zhang et al. (2000) utilized case studies, questionnaires, and interviews to examine how AD movies help visually impaired audiences have an equal viewing experience to those without visual impairments. Wang and Wang (2020) discussed issues such as the transformation between video rhetoric and text, as well as the preservation of the original film’s form and style. Yang et al. (2023) which focused on the reception of existing AD services in mainland China, revealed that “over interpretation, revealing the plot, subjective speculations are common practices in AD services in the Chinese mainland but are applauded by the participants for which helps them to understand the film.” Additionally, recent international efforts have provided a broader overview of AD in China. Tor-Carroggio and Casas-Tost (2020) outlined the profile of Chinese audio describers, while Tor-Carroggio and Rovira-Esteva (2019) investigated the satisfaction and experiences of Chinese AD users.

### 2.2 Audio description and presence

Presence is a multi-construct concept that extends the notion of “telepresence,” originally developed to describe human interaction with remote access technologies (Minsky, 1980). It can be defined as the “perceptual illusion of non-mediation” (Lombard and Ditton, 1997), the “illusion of being there” (Biocca, 1997, p. 18), or “the illusion of being located somewhere other than the physical environment” (Walczak and Fryer, 2018, p. 69). As a cognitive construct, presence influences how users feel and act as if the events they experience in virtual reality are happening in the real world (Cummings and Bailenson, 2015). This concept encompasses the psychological sense of immersion in any mediated environment (Fryer and Freeman, 2012b, p. 15).

As an access service, AD is crucial in enhancing audience engagement with audiovisual content. AD not only serves as a valuable resource to help users understand the material but also enables viewers to immerse themselves in the story, fully enjoying the cinematic experience. After all, the pursuit of entertainment is a key reason audiences are drawn to cinema (Davis et al., 2015). Numerous studies have demonstrated the positive effects of AD on media comprehension and the increased accessibility of visual content for visually impaired viewers (Schmeidler and Kirchner, 2001; Palomo, 2008; Chmiel and Mazur, 2012; Romero-Fresco and Fryer, 2013). However, only a few reception studies have explored the impact of AD on users’ emotional responses and their sense of presence.

Fryer and Freeman (2012a) investigated the impact of AD on the sense of presence in media, and found that participants with impaired sight reported higher levels of presence when watching stimuli with AD, compared to sighted people watching the same stimuli without AD. A comparison between standard AD and cinematic AD revealed that engagement scores were highest for the cinematic AD version among blind and partially sighted participants. In Bardini’s study (2020b), 45 blind and partially sighted Catalan viewers engaged with three different AD styles. The results indicated that both cinematic and narrative AD styles significantly enhanced the film experience, leading to greater satisfaction compared to conventional AD. Ramos Caro (2016) examined the emotional reception of AD by comparing objective and subjective descriptions, focusing on emotions such as fear, sadness, and disgust. The study found that subjective AD was more effective in conveying fear and sadness, and was positively received by users. Walczak and Fryer (2017) investigated the impact of creative AD on the sense of presence, and found that creative AD led to higher presence scores, with the majority of participants expressing a preference for it. Bardini (2017) tested three different AD styles—denotative, cinematic, and narrative—among visually impaired participants in Catalonia. The findings suggested that comprehension, enjoyment, and emotion were closely linked in the film experience and could be influenced by the style of AD employed. Hättich and Schweizer (2020) confirmed that AD, provided by an application, was a useful tool for people with sight loss, allowing them to immerse themselves and enjoy films as much as sighted people. Walczak’s (2017) study showed that creative AD led to higher levels of presence compared to standard AD. Additionally, AD narrated by a human voice generated significantly greater levels of presence for fiction than AD delivered by a synthetic voice.

## 3 An empirical study on the impact of film audio description style on the presence

This research builds on prior work but centers on Chinese blind and visually impaired audiences. It narrows the focus to the style of AD scripts—subjective versus objective. To minimize the potential influence of vocal delivery styles on the sense of presence, the study utilized identical artificially synthesized voices for the clips. The primary objective is to investigate how different AD styles affect users’ sense of presence and to evaluate user experiences associated with these styles. Ultimately, the study aims to determine whether subjective or objective AD leads to a greater sense of presence among Chinese visually impaired audiences.

### 3.1 Aim and hypothesis

The primary aim of this study is to assess whether there are differences in the presence elicited by two distinct AD versions of the same scenes: one that is more objective and another that is more subjective. The main hypothesis is that the subjective version will evoke a stronger sense of presence in Chinese visually impaired audiences, compared to the objective version.

### 3.2 Materials and stimuli

To examine the impact of subjective and objective AD styles on the sense of presence in Chinese visually impaired audiences, two romantic clips from *The Curious Case of Benjamin Button* (2008), a romantic fantasy drama directed by David Fincher, were selected. The film was introduced in China in 2009 and was dubbed into Mandarin. An audio-described version of the film is available in both China and the United Kingdom.

The first clip, lasting 12 min, is set in Murmansk in 1941. It depicts Benjamin’s infatuation with Elizabeth Abbott, the wife of the British trade mission’s chief minister. Their brief affair ultimately ends, leaving Benjamin heartbroken.

The second clip, 13 min in length, takes place in 1962. It portrays Daisy’s return to New Orleans and her reunion with Benjamin. Now at a similar physical age, they fall in love and decide to live together.

Two distinct AD scripts—one subjective and one objective—were employed in this study. As highlighted in the literature, British AD practices generally emphasize objective descriptions, whereas AD in China tends to adopt a more subjective approach. Accordingly, the subjective AD script was sourced from China, while the objective AD script was sourced from the UK. The latter was carefully translated into Chinese by the authors, ensuring both stylistic and content equivalence with the original script. Both scripts were used in their original forms without modification.

The two selected clips from the original Mandarin-dubbed movie were supplemented with objective and subjective AD scripts. Professionals from Gansu TV Station then produced them as test clips. To maintain consistency and minimize potential biases related to vocal delivery, both scripts were narrated using the same synthetic female voice. Table 1 presents excerpts from both versions for comparison, with the Chinese script translated into English for this article.

**Table 1:** Extracts from the AD scripts for *The Curious Case of Benjamin Button.*

Examples	Objective description	Subjective description
Eg.1 (Clip 1)	He’s found Elizabeth in the lobby of the hotel. She’s sitting on a sofa, a dressing gown over her nightdress, reading. Benjamin is in his dressing gown. He heads towards the kitchen and then turns back to her. Benjamin goes through to the kitchen. Elizabeth stares after him. She closes the magazine she was reading and gets to her feet. She peers into the kitchen where Benjamin is busy making the tea. She walks into the kitchen and Benjamin looks up. Elizabeth opens her mouth as if about to say something. Benjamin goes over to a worktop and picks up two Russian tea glasses in their holders.	Benjamin goes downstairs to the hotel lobby and sees Elizabeth sitting alone on the couch, reading a book. He greets her in a friendly way. Elizabeth is busy extinguishing the cigarette in her hand. Still reading. Benjamin turns and walks toward the kitchen. Elizabeth looks up at his back, and then Elizabeth slowly closes the book and stands up from the couch, watching him disappear into the kitchen. *Something unexpected always seems to happen to bored men and women on these quiet, cold nights*. Elizabeth walks towards the kitchen where Benjamin is filling the kettle with water.
Eg.2 (Clip 1)	The left rises. Elizabeth wears a fur coat and a hat with a broad brim, which casts her face in shadow. They gazed at each other. They arrived outside room 57 and he unlocked the door. He holds it open and turns to Elizabeth. She falls into his arms and they disappear into the room.	In the elevator, Elizabeth is waiting for him and they stand facing each other. *Each conveys adoration with their gaze. Though youth has passed, the passion of love still burns*. Exiting the elevator, Benjamin opens the door to his room and turns to hug Elizabeth. Finally, *Elizabeth manages to jump into Benjamin’s arms against all odds*.
Eg.3 (Clip 1)	He comes downstairs and finds the lobby deserted. He finds the dining room and the kitchen deserted too. He looks over at the bar. Benjamin takes a seat by the fire. As the night wears on, Benjamin drifts off to sleep. When he wakes, people are milling around the lobby.	Benjamin goes downstairs as usual to *prepare for his date with Elizabeth*, but no one is in the hall. He goes into the kitchen again, but Elizabeth is not there either. The hall is empty, with only the fire still burning in the fireplace. He walks over to the couch *where Elizabeth often sits and sits down, waiting for his sweetheart to arrive. But Elizabeth still does not appear, even as Benjamin eventually falls asleep on the couch.*
Eg.4(Clip 2)	He pauses on the porch. Daisy stands behind him. He turns to see her standing in the doorway opposite. Dressed in black, her hands knitted together at her waist. He stares at her for a moment. Then letting go of the screen door and purposefully walks across the porch to embrace her. She flings her arms around him with a big smile. They sit indoors.	Just as Benjamin is about to open the door and go upstairs, *he is surprised to find Daisy* in a black saree standing in the doorway opposite. The two stare at each other, *the surprise rendering them both speechless*. Benjamin takes a few steps forward and hugs Daisy in a flash. They go inside and sit opposite each other, feeling *as though they have a lot to say*.
Eg.5 (Clip 2)	Benjamin carries two of Daisy’s suitcases as they walk side by side through the silent shadowy corridors of the home. They enter a bedroom decorated with framed landscape paintings. Daisy puts her coat and handbag down on the double bed, which has tall, elaborately carved bedposts. She closes the door and turns around their eyes, meet across the dimly lit room. Step closer and Benjamin firmly clasped her torso. Daisy arches her back slightly as their lips meet in a slow.	*It is all so magical and unbelievable*. Together they walk through the living room towards Benjamin’s room. Once inside, Daisy closes and locks the door. *The parting, the loss, and the longing make their reunion tonday all the more passionate. They hug each other tightly and kiss sweetly. The two hearts press closer together*.
Eg.6 (Clip 2)	Benjamin and Daisy are silhouetted against the lilac sunset on the slowly drifting boat. Benjamin looks up to see a space rocket rise over billowing mountainous clouds on the horizon. Daisy rings out her wet hair and Benjamin playfully engulfs her in a stripy tone. They lounged together by these palm trees on golden sands. Standing beside her in the ocean, Benjamin watches Daisy as she raises her head from the still waters. Night. They made love on the deck. They dive off the prow of the boat as moonlight plays on the glittering surface of the sea. They swim gracefully beneath a brilliant full moon.	Benjamin sails along the Florida coast with Daisy in his sailboat, their joyful silhouettes gracing the shore at dusk, the beach in the afternoon, and the shallow bay at dawn. *They love each other with boundless passion, losing all sense of time as day and night blur in their fervent embrace. Immersed in the river of love, it feels as if nothing in the world could ever disrupt their bliss*.

### 3.3 A comparison of objective and subjective descriptions of the clips

Objective and subjective descriptions differ significantly in focus, language, perspective, and interpretation, as summarized in Table 2. Objective descriptions emphasize observable details and events, maintaining a neutral tone that allows the audience to interpret scenes independently. For example, in Clip 1, an objective description states, “Benjamin takes a seat by the fire. As the night wears on, Benjamin drifts off to sleep. When he wakes, people are milling around the lobby.” This straightforward, factual approach presents clear visual details without suggesting emotional interpretations, leaving room for the audience’s own understanding.

**Table 2:** The differences between objective descriptions and subjective descriptions.

Aspect	Objective description	Subjective description
Focus	Observable details and events.	Emotional interpretation and atmosphere.
Language	Factual, direct, neutral.	Evocative, expressive, figurative.
Perspective	Third-person, external viewpoint.	colored by a narrator’s or character’s emotional lens.
Interpretation	Leaves interpretation to the audience.	Suggests meaning, motivations, and relationships.

In contrast, subjective descriptions focus on emotional and atmospheric elements, offering interpretive insights into characters’ thoughts, motivations, or overall mood. For instance, in the same clip, a subjective description reads, “Benjamin sat down by the fire, waiting for his sweetheart to arrive. Until he fell asleep on the couch, Elizabeth still had not appeared.” This description conveys a sense of anticipation and longing, encouraging the audience to empathize with Benjamin’s emotional state.

The language and style further differentiate the two approaches. Objective descriptions rely on factual, direct, and neutral language, presenting events sequentially and focusing on concrete imagery. For instance, in Clip 2, an objective description states, “Benjamin and Daisy are silhouetted against the lilac sunset on the slowly drifting boat.” By contrast, subjective descriptions use rich, evocative language to enhance the emotional depth of a scene. The same scene was described subjectively as, “They loved each other with boundless passion, losing all sense of time as day and night blurred in their fervent embrace.” This romanticized version employs figurative devices such as metaphor and personification to immerse the audience in the characters’ experiences.

These two styles serve distinct storytelling purposes. Objective descriptions provide narrative neutrality, making them ideal for delivering information or setting the scene without bias. Subjective descriptions, on the other hand, engage the audience’s imagination and emotions, creating a more vivid and immersive storytelling experience.

### 3.4 Participants

Fifty participants (17 men and 33 women), aged 19 to 70 years (*M* = 35.5, SD = 14.12), were recruited from Lanzhou, Gansu Province, through the Gansu Association of the Visually Impaired, the Gansu Provincial Library, and personal networks. All participants were officially registered as visually impaired and reported their age of vision loss onset, which ranged from 2 to 51 years, with a mean of 14.36 years (SD = 11.19). Among them, 48% cited congenital causes of visual impairment, such as retinitis pigmentosa, cataracts, or glaucoma, while 52% reported acquired causes, including retinal detachment and progressive disorders. Educational attainment varied: 2% were illiterate, 6% had completed primary school, 36% middle school, 38% high school, and 18% college or higher. The severity of visual impairment ranged from extremely severe (28%) and severe (30%) to moderate (28%) and mild (14%). Ethical approval for the study was given by the first author’s institution.

### 3.5 Instruments

The Elicited Emotion Scale (Gross and Levenson, 1995) was used to assess the participants’ emotional experiences. Participants rated the intensity of their emotions experienced during the clip on a scale from 1 to 5, where 0 indicated “not feeling even the slightest bit of emotion” and 5 represented “the most you have ever felt in your life.” From the original 16 items, two relevant to this study were selected: Interest and Confusion.

The subjective feeling component was assessed using a validated self-report questionnaire commonly employed to measure emotional experiences. Participants’ immersion in the presented material was assessed through the ITC-Sense of Presence Inventory (Lessiter et al., 2001), which utilizes a 5-point Likert scale (1 = strongly disagree; 5 = strongly agree) to measure four dimensions of presence: sense of physical space, engagement, ecological validity, and negative effects. To mitigate participant fatigue, the short form of the inventory was administered, as has been utilized in several previous studies (Fryer and Freeman, 2014; Hammick and Lee, 2014; Walczak and Fryer, 2017).

For the positive subscales, three items were selected from each dimension: for a sense of physical space (“I felt I was visiting the places in the scenes,” “During the clip I had a sense of being in the scenes,” “I felt surrounded by the scenes”); for engagement (“I felt myself being drawn in,” “I lost track of time,” “I paid more attention to the scenes than to my own thoughts”); and for ecological validity (“the scenes seemed natural,” “the content seemed believable to me,” “I felt the environments were part of the real world”). A single item assessed negative effects: “I experienced sensations such as dizziness, disorientation, nausea, a headache, or tiredness.” For this study, the item “eye strain” was excluded from the negative sensations list.

### 3.6 Procedure

We adopted a dual approach that incorporates both questionnaires and interviews. This methodology allows us to complement quantitative data with qualitative insights.

The individual questionnaires primarily consisted of a Likert scale and short questions, while interviews were conducted in a group format. Participants were organized into focus groups of 5 individuals, each engaged in an experimental session lasting about 1 h. Each participant had a volunteer to assist in completing the questionnaire and interview. Before the session, the aims of the study were explained and informed consent forms were signed.

At the beginning of the session, each participant was provided with a small bottle of water and some cakes, fostering an informal atmosphere. We began with sociodemographic questions to facilitate introductions among participants. Following this, participants watched both clips, each presented in a different AD style on a laptop without headphones. The order of the clips and the AD styles were counterbalanced throughout the sample. To help viewers better understand the movie clips and follow the plot, each movie clip is preceded by a short audio introduction to the plot before the clip. Participants listened to one of the AD versions and subsequently completed the questionnaire. To mitigate potential contamination effects that could skew the results, participants answered the questionnaire items individually, using a specially designed one-rod abacus with five beads.

Discussion occurred after each section of the questionnaire. Participants were asked to share their experiences and preferences regarding the descriptions and were encouraged to provide additional comments.

## 4 Results and discussion

### 4.1 The impact of AD styles on presence

To examine the influence of subjective versus objective audio description styles on presence among visually impaired audiences, we conducted paired t-tests (Tables 3, 4) and effect size calculations (Table 5) using SPSS. The results show significant differences between the two styles, suggesting that subjective audio descriptions are more effective in enhancing key dimensions of user experience, including interest, clarity, spatial awareness, engagement, and ecological validity. These dimensions are critical in fostering a sense of presence among visually impaired audiences, enabling a more immersive and satisfying film experience.

**Table 3:** Paired sample statistics for AD styles and presence.

Paired sample statistics		Mean	N	SD	SE
Paired 1	Interest_subjective_style	4.34	50	1.081	0.153
	Interest_objective_styles	3.96	50	1.087	0.154
Paired 2	Confusion_subjective_style	1.44	50	0.861	0.122
	Confusion_objective_styles	2.20	50	1.245	0.176
Paired 3	Sense_of_physical_space_subjective_style	3.9933	50	1.03343	0.14615
	Sense_of_physical_space_objective_styles	3.1667	50	1.17466	0.16612
Paired 4	Engagement_subjective_style	3.9733	50	0.90311	0.12772
	Engagement_objective_styles	3.3800	50	1.15079	0.16275
Paired 5	Ecological_Validity_subjective_style	4.0600	50	0.98444	0.13922
	Ecological_Validity_objective_styles	3.2067	50	1.16231	0.16438
Paired 6	Negative_effects_subjective_style	1.08	50	0.444	0.063
	Negative_effects_objective_styles	1.22	50	0.764	0.108

**Table 4:** Results of paired sample t-test for AD styles and presence.

Pair		Paired Differences					t	df	Significance Levels	
		Mean Diff.	SD	SE	95% CI				One-tailed P	Two-tailed P
					LL	U L				
Pair 1	Interest_subjective_style Interest_objective_styles	0.380	1.210	0.171	0.036	0.724	2.220	49	0.016	0.031
Pair 2	Confusion_subjective_style Confusion_objective_styles	−0.760	1.506	0.213	−1.188	−0.332	−3.569	49	0.000	0.001
Pair 3	Sense_of_physical_space_subjective_style Sense_of_physical_space_objective_styles	0.82667	1.54632	0.21868	0.38721	1.26613	3.780	49	0.000	0.000
Pair 4	Engagement_subjective_style Engagement_objective_styles	0.59333	1.43378	0.20277	0.18586	1.00081	2.926	49	0.003	0.005
Pair5	Ecological_Validity_subjective_style Ecological_Validity_objective_styles	0.85333	1.48791	0.21042	0.43047	1.27619	4.055	49	0.000	0.000
Pair 6	Negative_effects_subjective_style Negative_effects_objective_styles	−0.140	0.904	0.128	−0.397	0.117	−1.095	49	0.139	0.279

**Table 5:** Results of effect size for paired samples for AD styles and presence.

Pair			Standardized measure^a^	Estimate	95% confidence interval	
					Lower limit	Upper limit
Pair 1	Interest_subjective_style Interest_objective_styles	Cohen d	1.210	0.314	0.028	0.596
		Hedges’ g	1.229	0.309	0.028	0.587
Pair 2	Confusion_subjective_style Confusion_objective_styles	Cohen d	1.506	−0.505	−0.797	−0.208
		Hedges’ g	1.529	−0.497	−0.785	−0.205
Pair 3	Sense_of_physical_space_subjective_style Sense_of_physical_space_objective_styles	Cohen d	1.54632	0.535	0.236	0.829
		Hedges’ g	1.57050	0.526	0.232	0.816
Pair 4	Engagement_subjective_style Engagement_objective_styles	Cohen d	1.43378	0.414	0.123	0.701
		Hedges’ g	1.45621	0.407	0.121	0.690
Pair 5	Ecological_Validity_subjective_style Ecological_Validity_objective_styles	Cohen d	1.48791	0.574	0.272	0.870
		Hedges’ g	1.51118	0.565	0.267	0.857
Pair 6	Negative_effects_subjective_style Negative_effects_objective_styles	Cohen d	0.904	−0.155	−0.433	0.125
		Hedges’ g	0.918	−0.153	−0.426	0.123

a The denominator used for effect size estimation is the standard deviation of the mean difference.

Cohen’s d uses the standard deviation of the mean difference.

Hedges’ g uses the standard deviation of the mean difference with an adjustment factor.

#### 4.1.1 Interest

The subjective style yielded a higher mean score of 4.34 compared to 3.96 for the objective style, with a significant *t*-value (t(49) = 2.220, *p* = 0.031). The large effect size (Cohen’s *d* = 1.210, Hedges’ *g* = 1.229) confirms that subjective descriptions are perceived as more engaging by viewers. This suggests that the subjective style may help hold the audience’s attention by incorporating interpretive elements.

#### 4.1.2 Confusion

Confusion scores were significantly lower for the subjective style (*M* = 1.44) than for the objective style (*M* = 2.20), with t(49) = −3.569 and *p* < 0.001, indicating greater clarity in subjective descriptions. The large effect size (Cohen’s *d* = 1.506, Hedges’ *g* = 1.529) supports the idea that subjective descriptions reduce ambiguity and cognitive load, potentially due to additional contextual cues and interpretive language, such as expressions of subjective judgments and comments.

#### 4.1.3 Sense of physical space

Participants reported a stronger sense of physical space with the subjective style (*M* = 3.99) compared to the objective style (*M* = 3.17), showing a significant difference (t(49) = 3.780, *p* < 0.001). The large effect size (Cohen’s *d* = 1.546, Hedges’ *g* = 1.571) suggests that the subjective approach more effectively conveys spatial dimensions, likely enhancing the immersive experience for visually impaired viewers.

#### 4.1.4 Engagement

The subjective style also significantly outperformed the objective style in terms of engagement, with mean scores of 3.97 and 3.38, respectively (t(49) = 2.926, *p* = 0.005). The large effect size (Cohen’s *d* = 1.434, Hedges’ *g* = 1.456) suggests that subjective descriptions foster a deeper emotional and cognitive connection with the content.

#### 4.1.5 Ecological validity

The subjective style was perceived as more realistic, scoring higher in ecological validity (*M* = 4.06) than the objective style (*M* = 3.21), with a significant t-value (t(49) = 4.055, *p* < 0.001). The large effect size (Cohen’s *d* = 1.488, Hedges’ *g* = 1.511) further supports the notion that subjective descriptions offer a more authentic and believable experience for viewers, contributing to the overall immersion.

#### 4.1.6 Negative effects

While negative effect scores were low for both styles, the objective style scored slightly higher (*M* = 1.22) than the subjective style (*M* = 1.08). The t-test did not yield a significant difference (t(49) = −1.095, *p* = 0.279), and the effect size (Cohen’s *d* = 0.904, Hedges’ *g* = 0.918) was relatively small. This finding suggests that neither style is associated with substantial adverse effects, though the subjective style may be marginally less likely to induce negative responses.

The large effect sizes observed in this study highlight the substantial practical significance of AD style. These results suggest that AD style extends beyond mere statistical differences, profoundly influencing the media experience for visually impaired audiences.

In practical terms, adopting a more subjective approach to AD can significantly enhance emotional engagement, fostering a deeper connection with the content. Through interpretive cues and richer contextual information, subjective AD not only reduces cognitive load and enhances clarity but also amplifies spatial and ecological presence, creating a more immersive and enjoyable viewing experience. This, in turn, can lead to higher viewer retention, increased satisfaction, and greater active engagement with audiovisual media.

These improvements also have critical implications for media accessibility. Subjective AD can contribute to a more inclusive media environment, enabling visually impaired viewers to navigate and enjoy content more independently. Furthermore, these advancements could promote more positive attitudes toward inclusive cultural participation and broader accessibility initiatives. As AD services continue to develop, especially in regions like China, these findings underscore the importance of not only prioritizing informational accuracy but also tailoring AD to align with audience preferences and enhance presence.

In addition to the quantitative analysis, qualitative data from focus group discussions further illuminated participants’ perceptions of the two AD styles. Participants were asked to share their experiences and preferences regarding subjective and objective descriptions and were encouraged to provide any additional comments.

Many participants expressed a preference for the subjective style, noting that it felt more emotionally engaging. One participant remarked, “With the subjective descriptions, I could truly sense the characters’ emotions, which made me feel more connected to the story as if I were living it alongside them.” Another shared, “The objective description was clear, but I did not feel as engaged with the scene.”

While subjective descriptions were seen as more immersive, several participants noted that they could sometimes be confusing if they were too rich in detail or overly emotional. One participant explained, “The subjective descriptions sometimes added too much emotion, which made it harder to understand what was actually happening.” However, others found the subjective style clearer, with one participant commenting, “The additional context provided in the subjective style, particularly the interpretation and subjective judgments and comments, helped me better understand the characters and follow the story more easily.”

When discussing spatial awareness, participants were split. Some felt that the subjective style provided a clearer sense of space, especially in scenes with strong emotional or atmospheric elements. One participant noted, “In scenes where Benjamin and Daisy were together, I could picture the setting much better. It felt like I was in the scene with them.” However, a few participants felt that excessive emotional detail in the subjective descriptions occasionally detracted from their understanding of spatial details. One participant noted, “I liked the subjective descriptions, but sometimes I missed knowing exactly where they were or what they were doing physically.”

The subjective style was widely praised for offering a more realistic and relatable experience. Many participants commented on how the descriptions made the film feel more authentic, with one saying, “The subjective descriptions felt real like I was experiencing the emotions and atmosphere of the scene myself.” However, a few participants expressed that the heightened emotional language could sometimes overshadow the realism of the setting. One participant mentioned, “It was realistic, but at times it felt a bit too dramatic, which made me lose touch with the actual environment.”

Both quantitative analysis and qualitative data from focus group discussions indicate that subjective audio description styles have a notable advantage in creating an engaging and immersive experience for visually impaired audiences. By enhancing clarity, spatial awareness, and ecological validity, subjective descriptions support a stronger sense of presence. Given the consistently large effect sizes, particularly in interest and spatial perception, the findings suggest that subjective descriptions are especially beneficial in contexts requiring emotional engagement and clear spatial understanding. Future studies could examine whether these benefits hold across different genres and settings, potentially extending the applicability of subjective audio descriptions in media accessibility initiatives.

### 4.2 Exploratory analyses on the moderating effects of age and onset of visual impairment

To explore whether individual demographic factors moderate the effects of AD style (subjective vs. objective) on presence, two repeated measures ANOVAs were conducted, examining age and onset of visual impairment (congenital vs. acquired) as potential moderators.

First, the analysis examining age revealed no significant interaction effects across any of the six dimensions. Specifically, for interest, the AD style × age interaction was non-significant, *F*(1,48) = 0.538, *p* = 0.927, indicating that the influence of AD style on interest was consistent across age groups. Similar null interactions were observed for confusion (*F* = 0.498, *p* = 0.957), negative effects (*F* = 0.816, *p* = 0.696), sense of physical space (*F* = 0.657, *p* = 0.851), ecological validity (*F* = 1.202, *p* = 0.333), and engagement (*F* = 0.695, *p* = 0.816). Additionally, age showed no significant main effects on any dimension (all ps > 0.05). These results suggest that age does not moderate the relationship between AD style and audience presence.

In contrast, the analysis examining onset of visual impairment revealed significant interaction effects for sense of physical space and engagement. For sense of physical space, a significant interaction emerged, F(1,48) = 5.413, *p* = 0.024. Ratings for the subjective AD were similar between groups (congenital: *M* = 3.93, SD = 0.96; acquired: *M* = 4.05, SD = 1.11); however, scores for the objective AD were notably lower in the acquired group (*M* = 2.76, SD = 1.21) than in the congenital group (*M* = 3.61, SD = 0.98). This suggests that individuals with acquired vision loss may benefit more from the spatial elaboration afforded by subjective AD.

Similarly, for engagement, a significant interaction was observed, F(1,48) = 7.778, *p* = 0.008. Both groups responded positively to the subjective AD (congenital: *M* = 3.82, SD = 0.91; acquired: *M* = 4.12, SD = 0.89), but engagement dropped considerably under the objective AD in the acquired group (*M* = 3.01, SD = 1.28), compared to the congenital group (*M* = 3.78, SD = 0.84). This indicates a stronger reliance on subjective cues for emotional engagement among individuals with acquired vision loss.

No significant interaction effects or main effects were found for the remaining dimensions—interest, confusion, ecological validity, and negative effects—implying that AD style influenced these aspects similarly across onset groups. These findings underscore the importance of considering the onset of visual impairment in designing AD content. The enhanced effectiveness of subjective AD in fostering spatial and emotional presence among individuals with acquired vision loss may reflect differences in perceptual habits or previous audiovisual experiences. Future studies are warranted to further investigate these mechanisms and refine AD practices for diverse user populations.

### 4.3 The relationship between AD style preference and gender

SPSS analysis revealed a clear preference for the subjective AD, with 80% of participants favoring it over the objective AD, which was preferred by only 20%. Contrary to the findings of Walczak and Fryer (2017), a Chi-square test (Table 6) in our study indicated no statistically significant relationship between gender and preferences for audio description styles. The Pearson Chi-square yielded a *p*-value of 0.654, greater than the conventional significance threshold of 0.05, indicating no significant association between gender and AD style preference. Fisher’s Exact Test corroborated this result, showing a two-tailed exact significance of 0.717 and a one-tailed exact significance of 0.461, further confirming that there is no significant association between gender and AD style preferences in this sample.

**Table 6:** Chi-Square test results for the relationship between AD style preference and gender.

Test	Value	Degrees of freedom	Asymptotic significance (Two-sided)	Exact significance (Two-sided)	Exact Significance (One-sided)
Pearson Chi-Square	0.201^a^	1	0.654		
Continuity correction^b^	0.006	1	0.941		
Likelihood Ratio	0.197	1	0.657		
Fisher’s Exact Test				0.717	0.461
Linear-by-Linear Association	0.197	1	0.658		
Number of Valid Cases	50				

a One cell (25.0%) has an expected count of less than 5. The minimum expected count is 3.40.

b Calculated only for 2×2 tables.

### 4.4 The impact of the degree of sight loss on the presence

A one-way ANOVA was conducted in SPSS to examine differences in various aspects of presence—sense of physical space, engagement, and ecological validity—across groups with varying levels of sight ability under both subjective and objective AD styles.

The results, summarized in Table 7, showed no statistically significant differences between groups (all *p*-values > 0.05). This finding contrasts with the results of Walczak and Fryer (2017), as no aspect of presence (sense of physical space, engagement, or ecological validity) showed significant variation by sight ability in this sample, regardless of AD style. This suggests that, within this sample, sight ability does not significantly impact presence under either subjective or objective audio description conditions.

**Table 7:** One-way ANOVA results for the relationship between degree of sight loss and presence.

ANOVA		Sum of squares	df	Mean square	F	*p*
Sense_of_physical_space_subjective_style	Between groups	4.485	3	1.495	1.437	0.244
	Within groups	47.846	46	1.040		
	Total	52.331	49			
Sense_of_physical_space_objective_styles	Between groups	5.130	3	1.710	1.259	0.300
	Within groups	62.481	46	1.358		
	Total	67.611	49			
Engagement_subjective_style	Between groups	2.083	3	0.694	0.843	0.477
	Within groups	37.881	46	0.824		
	Total	39.964	49			
Engagement_objective_styles	Between groups	1.092	3	0.364	0.263	0.852
	Within groups	63.799	46	1.387		
	Total	64.891	49			
Ecological_Validity_subjective_style	Between groups	1.320	3	0.440	0.438	0.727
	Within groups	46.167	46	1.004		
	Total	47.487	49			
Ecological_Validity_objective_styles	Between groups	1.420	3	0.473	0.336	0.799
	Within groups	64.777	46	1.408		
	Total	66.198	49			

However, differences in sample sizes across groups limit the reliability and interpretability of these results. Small group sizes, particularly in the “moderate” (*N* = 9) and “mild” (*N* = 6) groups, resulted in wide confidence intervals, high standard errors, and potential distortions in random-effects estimates. These factors reduce the statistical power of the analysis, potentially contributing to the lack of significant findings. The “extremely severe” group, with *N* = 22, was the largest, while the “severe” group had *N* = 13, illustrating the variability in sample sizes among groups. To improve reliability in future research, increasing sample sizes for underrepresented groups may be beneficial. Additionally, exploring potential interaction effects or other factors that could influence presence perceptions among visually impaired audiences could provide further insight.

### 4.5 The impact of the degree of education on the presence

A one-way ANOVA was conducted in SPSS focusing on the effects of education on different aspects of presence (sense of physical space, engagement, and ecological validity) for both subjective and objective AD styles. The results (Table 8) show educational level does not seem to produce statistically significant differences in the sense of presence, whether measured by sense of physical space, engagement, or ecological validity, across subjective and objective AD styles.

**Table 8:** One-way ANOVA results for the relationship between education and presence.

ANOVA		Sum of squares	df	Mean square	F	p
Sense_of_physical_space_subjective_style	Between groups	5.705	4	1.426	1.376	0.257
	Within groups	46.626	45	1.036		
	Total	52.331	49			
Sense_of_physical_space_objective_styles	Between groups	8.122	4	2.031	1.536	0.208
	Within groups	59.489	45	1.322		
	Total	67.611	49			
Engagement_subjective_style	Between groups	1.898	4	0.475	0.561	0.692
	Within groups	38.066	45	0.846		
	Total	39.964	49			
Engagement_objective_styles	Between groups	6.830	4	1.708	1.323	0.276
	Within groups	58.061	45	1.290		
	Total	64.891	49			
Ecological_Validity_subjective_style	Between groups	3.769	4	0.942	0.970	0.433
	Within groups	43.718	45	0.972		
	Total	47.487	49			
Ecological_Validity_objective_styles	Between groups	8.332	4	2.083	1.620	0.186
	Within groups	57.866	45	1.286		
	Total	66.198	49			

To explore whether educational background influenced participants’ preferences for AD style (subjective vs. objective), a chi-square test of independence was conducted. The results revealed no significant association between education level and AD style preference, χ^2^(4) = 3.656, *p* = 0.455. Of the 50 participants, 40 preferred the subjective AD, and 10 preferred the objective AD, with no discernible trend across educational groups.

However, the chi-square test assumptions were violated, as 70% of the cells had expected counts below 5—particularly among participants with lower education levels. To account for this, a Fisher–Freeman–Halton exact test was employed, yielding similarly non-significant results (*p* = 0.362), corroborated by a Monte Carlo simulation (99% CI: [0.349, 0.374]).

These findings suggest that within the current sample, education background did not significantly influence AD style preference. Nevertheless, the imbalanced distribution of education levels limits the generalizability of the results. Future research with more stratified samples may illuminate whether and how educational background interacts with cognitive processing or emotional engagement in response to different AD styles, potentially enriching our understanding of user diversity in AD reception.

### 4.6 Practical implications for AD production and policy in China

The findings of this study provide meaningful insights for the development of AD production practices and policy-making in China, where standardized national AD guidelines have yet to be established. The clear preference for subjective AD—particularly among individuals with acquired vision loss—suggests that greater flexibility in AD scriptwriting should be encouraged, allowing for more interpretive and emotionally expressive content. This contrasts with the objective style traditionally emphasized in many international AD standards.

For AD producers and scriptwriters, these results highlight the importance of integrating culturally resonant and emotionally engaging narrative elements into AD scripts. Rather than strictly adhering to a factual delivery, AD in the Chinese context may benefit from incorporating subjective descriptions that reflect character emotions and interpersonal dynamics, thereby enhancing audience presence and engagement. Furthermore, incorporating feedback from visually impaired users into the AD creation process could help tailor content to actual audience needs and preferences.

At the policy level, efforts to establish national AD guidelines in China could consider adopting a balanced approach—recognizing the value of both objective and subjective styles depending on the context and audience profile. Such a framework would allow for stylistic diversity while maintaining accessibility and consistency. Policies that support audience-centered AD production, including funding mechanisms and regulatory incentives, could further promote high-quality, user-responsive content.

Additionally, the genre of the film may influence how viewers respond to different AD styles. This study used a romantic drama, where emotional resonance was central to the viewing experience. Subjective AD likely enhanced this emotional engagement by conveying internal states and relational nuances. However, whether the same effects would be observed in action films, comedies, documentaries, or other genres—where narrative pacing and tonal expectations differ—remains an open question. Future research should explore genre-specific AD strategies to ensure that the descriptive style aligns with both genre conventions and audience expectations.

## 5 Conclusion

This study highlights the significant advantages of adopting subjective audio description styles in enhancing the sense of presence among Chinese visually impaired audiences. By incorporating interpretive and emotional elements, subjective ADs demonstrated improvements in key dimensions of presence, including spatial awareness, engagement, ecological validity, and overall clarity, compared to objective AD styles. These findings underscore the potential of subjective ADs to foster deeper emotional and cognitive connections with audiovisual content, leading to a more immersive and enriching viewing experience.

Importantly, the study found that demographic factors such as degree of sight loss, gender, and education level did not significantly moderate the effects of AD style on presence, suggesting the broad applicability of subjective ADs across diverse groups within the Chinese visually impaired population. This universality underscores the relevance of subjective AD as a promising approach for enhancing media accessibility and inclusivity.

Future research should build on these findings by exploring the effectiveness of subjective ADs across different film genres, cultural contexts, and larger demographic samples. Furthermore, longitudinal studies could investigate the sustained impact of subjective ADs on user preferences and media engagement over time. By addressing these questions, researchers and practitioners can refine audio description practices to better meet the needs of Chinese visually impaired audiences, ultimately contributing to a more inclusive media landscape.

## Data Availability

The original contributions presented in the study are included in the article/supplementary material, further inquiries can be directed to the corresponding author.
